# Multimodal deep learning with MUF-net for noninvasive WHO/ISUP grading of renal cell carcinoma using CEUS and B-mode ultrasound

**DOI:** 10.3389/fphys.2025.1558997

**Published:** 2025-03-07

**Authors:** Yixin Zhu, Ji Wu, Qiongxian Long, Yan Li, Hao Luo, Lu Pang, Lin Zhu, Hui Luo

**Affiliations:** ^1^ Department of Ultrasound, Peking University Shenzhen Hospital, Shenzhen, Guangdong, China; ^2^ Department of Urology, The Affiliated Nanchong Central Hospital of North Sichuan Medical College, Nanchong, Sichuan, China; ^3^ Department of Pathology, The Affiliated Nanchong Central Hospital of North Sichuan Medical College, Nanchong, Sichuan, China; ^4^ Department of Ultrasound, The Affiliated Nanchong Central Hospital of North Sichuan Medical College, Nanchong, Sichuan, China; ^5^ Department of Clinical and Research, Shenzhen Mindray Bio-medical Electronics Co., Ltd., Shenzhen, China; ^6^ Department of Ultrasound, The Second Clinical Medical College, Jinan University (Shenzhen People’s Hospital), Shenzhen, Guangdong, China

**Keywords:** renal tumor, artificial intelligence, classification, deep learning, WHO/ISUP grading system, contrast-enhanced ultrasound

## Abstract

**Objective:**

This study aimed to develop and validate a multimodal deep learning model that utilizes preoperative grayscale and contrast-enhanced ultrasound (CEUS) video data for noninvasive WHO/ISUP nuclear grading of renal cell carcinoma (RCC).

**Methods:**

In this dual-center retrospective study, CEUS videos from 100 patients with RCC collected between June 2012 and June 2021 were analyzed. A total of 6,293 ultrasound images were categorized into low-grade (G1-G2) and high-grade (G3-G4) groups. A novel model, the Multimodal Ultrasound Fusion Network (MUF-Net), integrated B-mode and CEUS modalities to extract and fuse image features using a weighted sum of predicted weights. Model performance was assessed using five-fold cross-validation and compared to single-modality models. Grad-CAM visualization highlighted key regions influencing the model’s predictions.

**Results:**

MUF-Net achieved an accuracy of 85.9%, outperforming B-mode (80.8%) and CEUS-mode (81.8%, *P* < 0.05) models. Sensitivities were 85.1%, 80.2%, and 77.8%, while specificities were 86.0%, 82.5%, and 82.7%, respectively. The AUC of MUF-Net (0.909, 95% CI: 0.829-0.990) was superior to B-mode (0.838, 95% CI: 0.689-0.988) and CEUS-mode (0.845, 95% CI: 0.745-0.944). Grad-CAM analysis revealed distinct and complementary salient regions across modalities.

**Conclusion:**

MUF-Net provides accurate and interpretable RCC nuclear grading, surpassing unimodal approaches, with Grad-CAM offering intuitive insights into the model’s predictions.

## 1 Introduction

About 80% ∼ 90% of renal malignancies are RCC, and there are three main pathological types of RCC: ccRCC (60% ∼ 85%), pRCC, and chromophobe renal cell carcinoma (chRCC, 4% ∼ 5%). Histological types and nuclear grading are important prognostic risk factors in RCC(Galtung et al., 2022; Inamura, 2017). Traditional imaging to diagnose RCC pathologic subtypes is challenging and does not differentiate its nuclear grading. The 2016 edition of WHO/ISUP categorizes RCC into four grades, and the grading criteria and related terminology are described in more detail than the previous Fuhrman grading criteria, with better reproducibility and clinical significance (Moch et al., 2016b). The WHO/ISUP grading system applies to ccRCC and pRCC, and RCC nuclear classification is associated with clinical treatment strategies and patient prognosis (Galtung et al., 2022). The study showed that the 5-year survival rates of patients with RCC histologic grades G1, G2, and G3-G4 were 89%, 65%, and 46%, respectively (Galtung et al., 2022; Tsui et al., 2000; Leclercq et al., 2007).

Lower-grade tumors represent cancer cells that more closely resemble normal cells. G1 and G2 tumors tend to grow slowly, have better differentiation, and spread less, making patients eligible for nephron-sparing surgery. In contrast, cancer cells in G3 and G4 tend to grow quickly, are poorly differentiated, and spread rapidly, with a higher recurrence rate and worse outcomes after surgery. As a result, more aggressive radical nephrectomy and stricter postoperative monitoring are usually required (Moch et al., 2016a; Novara et al., 2007). However, to obtain pathologic nuclear grading, renal biopsy or pathological examination after tumor resection is commonly used in clinical practice. Notably, recent studies have shown that renal puncture biopsy is less accurate in determining RCC nuclear grading (62.5%–83%) with a tendency to underestimate (Perrino et al., 2018; Bernhard et al., 2015). Additionally, biopsy procedures might elevate the risk of complications, including bleeding, infection, and even the potential hazard of tumor rupture (Wang et al., 2009). Such invasive procedures have the potential to inflict considerable physical and psychological distress on patients, while concurrently imposing a significant economic burden on both the families involved and society at large. RCC is one of the few malignancies currently known to achieve significantly reduced long-term recurrence through early surgical intervention. Early, non-invasive, and accurate subtyping of RCC is crucial for minimizing inter-observer variability in pathologic diagnoses. This approach plays a pivotal role in assisting clinicians with real-time, individualized, and precise therapeutic strategies, optimizing intervention timing, and maximizing patient outcomes. Deep learning has demonstrated promising accuracy in noninvasively distinguishing benign renal tumors from RCC using ultrasound and MR imaging across multi-institutional datasets. Numerous studies have reported successful applications of deep learning for classifying benign and malignant renal tumors (Xi et al., 2020; Zhu et al., 2022). However, research on its use for noninvasive RCC grading remains limited. Previous research has employed traditional machine learning techniques using CT/MRI texture features for the nuclear grading of RCC, yielding promising preliminary results (Cui et al., 2020; Lin et al., 2019). These research findings have highlighted the potential of applying machine learning and deep learning to investigate the relationship between medical imaging data and the pathological histology of renal malignant tumors. They provide the impetus for addressing the more complex task of nuclear grading in RCC within ongoing research efforts. However, this field is still in its early stages, and the number of studies remains limited. There is significant variability in model accuracy and reproducibility across different institutions, and it is still unclear whether CEUS presents any distinct advantages over CT/MRI. Further in-depth investigation into this area is necessary.

To address challenges in noninvasive RCC grading, we explore the feasibility of applying transfer learning with the Multimodal Ultrasound Fusion Network (MUF-Net) from our preliminary research (Zhu et al., 2022), which has exhibited exemplary performance in classifying the malignancy of renal solid tumor. This study aims to evaluate the potential of transfer learning to further optimize MUF-Net for robust WHO/ISUP grading of RCC across diverse datasets, providing a scalable and effective solution for clinical applications.

## 2 Materials and methods

### 2.1 Study design

**FIGURE 1:**
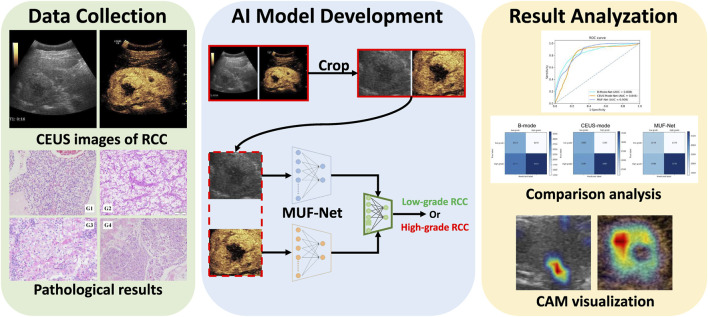
The overall workflow of this study.

In this dual-center retrospective study, a total of 6,293 ultrasound images were obtained from CEUS videos of 100 patients diagnosed with renal malignancies. The MUF-Net model facilitated the automated extraction of image features from both modalities and performed a weighted summation based on two predicted weights, thereby fusing the multi-modal features. The data were randomly split into a training cohort (80%) and a test cohort (20%). To prevent overfitting and improve performance, data augmentation techniques were employed to expand the training cohort. Model performance was evaluated using a five-fold cross-validation procedure, comparing MUF-Net to single-modality models. Grad-CAM was generated to visualize the salient regions influencing the RCC nuclear grading predictions. Figure 1 illustrates the study workflow.

### 2.2 Data collection

A retrospective collection of 100 cases of patients diagnosed with RCC through postoperative histopathology from June 2012 to June 2021 was conducted at two medical centers. The clinical data included gender, age, BMI, clinical symptoms (e.g., lumbar discomfort, hematuria), laboratory indicators (white blood cell count, red blood cell count, hemoglobin, platelet count, albumin, alkaline phosphatase, lactate dehydrogenase, total calcium), tumor size, pathological type, and other relevant information. All pathological diagnoses included WHO/ISUP grading results. The study was approved by the ethics review committees of both hospitals. All patients had complete greyscale ultrasound and CEUS imaging data.

The inclusion criteria are as follows: (1) Postoperative histopathological findings confirmed the diagnoses of ccRCC and pRCC. Pathology reviews were conducted by pathologists with over 8 years of clinical experience, using the WHO/ISUP grading system for histopathological evaluation to determine the final results of RCC nuclear grading. (2) All patients underwent preoperative grayscale ultrasound and CEUS, with more than 2–3 min of complete and clear CEUS video data. (3) Prior to surgery, the patients received no interventional, pharmacological, or other antitumor treatments. (4) The renal tumor was solid. (5) Patients had complete clinical data.

The exclusion criteria are as follows: (1) The histological type was chRCC. (2) Clinicopathological information was incomplete. (3) CEUS was not performed, or the quality of the CEUS imaging was poor, failing to capture the entire renal tumor. The retained CEUS video duration was too short to capture the characteristics of tumor enhancement and washout. (4) Pathological stage ≥ T2b, maximum tumor diameter >10 cm, or tumor involvement of the perirenal area, including lymph node or distant metastases.

### 2.3 Ultrasound examination

CEUS examinations were performed using the following three ultrasound systems: LOGIQ E9 (GE Healthcare, United States), Resona7 (Mindray, Shenzhen, China), and IU22 (Philips, Netherlands), all equipped with 1.0–5.0 MHz convex probes. CEUS was conducted using ultrasound machines with contrast-specific software, administering a bolus of 1.0–1.2 mL of microbubble contrast agent (SonoVue; Bracco, Milan, Italy) via the antecubital vein, followed by 5.0 mL of normal saline. Each CEUS digital video lasted at least 3–5 min.

### 2.4 Data annotation and preprocessing

CEUS videos were annotated using Pair annotation software (v2.6, RayShape Medical Technology, Shenzhen, China). In each CEUS video, approximately 50–60 images were selected from the cortical and medullary phases. A senior radiologist classified the tumors as either benign or malignant and annotated their locations in each selected image using bounding boxes. Based on the bounding boxes, these images were cropped into smaller regions of interest (ROIs) to exclude non-tumor areas. After the dataset was fully labeled in 100 cases, a total of 6,293 images were obtained: 3,573 images for the low-grade group (G1-G2) from 74 cases, and 2,720 images for the high-grade group (G3-G4) from 26 cases.

### 2.5 Deep neural network structure

**FIGURE 2:**
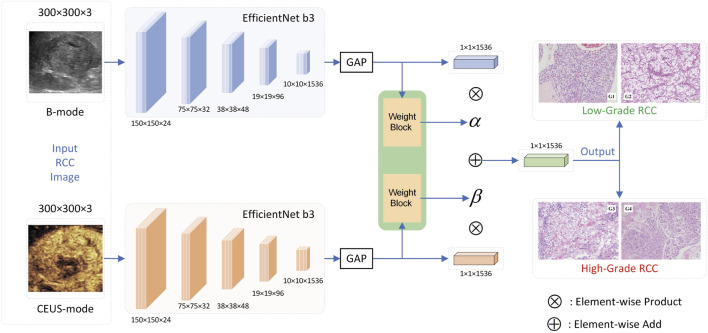
The overall architecture of MUF-Net. GAP: Global Average Pooling.

In our prior research (Zhu et al., 2022; Li et al., 2023), the MUF-Net model we developed, which fuses B-mode and CEUS-mode images features, has significantly improved the classification performance for benign versus malignant solid renal tumors. Therefore, we hypothesize that MUF-Net can leverage both B-mode and CEUS data to classify different histological subtypes of RCC, potentially outperforming single-modality models. To construct MUF-Net, we used a similar approach as in our previous work, training and testing several mainstream neural network algorithms and comparing their performance. The overall architecture of MUF-Net is shown in Figure 2. Ultimately, EfficientNet-b3 was selected as the backbone for MUF-Net due to its superior performance. The input size of the backbone was 300 × 300 × 3, and after five downsampling blocks, the output size became 10 × 10 × 1,536. To decrease the network’s parameters and avoid overfitting, we implemented a global average pooling layer, which reduced the output feature maps of each backbone from 10 × 10 × 1,536 to 1 × 1 × 1,536. Following this, we combined the features from the two modalities. Recognizing that each modality’s features in a sample might have varying impacts on the final prediction, we developed two attention blocks with shared weights to generate adaptive weights α and β for the fusion of modalities. The feature maps from the two modalities were then weighted and summed according to these adaptive weights, resulting in a fused feature map of 1 × 1 × 1,536. Ultimately, a fully connected layer and a SoftMax layer were used to provide the classification result.

In this study, we applied MUF-Net for transfer learning to differentiate between low-grade (G1-G2) and high-grade (G3-G4) RCC groups. We compared its performance with single-modality EfficientNet-b3 models based solely on B-mode or CEUS data to comprehensively evaluate the model’s efficacy. MUF-Net initially extracts image features from the tumor regions using two shared-weight EfficientNet-b3 networks, each processing one of the two modalities. The Adaptive Weight Module then predicts the importance of each modality, and the features are weighted and combined accordingly. Finally, the RCC grade is predicted based on the fused features.

### 2.6 Modeling and evaluation

We designed a series of comparative experiments to evaluate the performance of different models in the histological grading of RCC. All models were deployed on the same dataset with uniform parameter configurations and were tested under consistent environments. To rigorously validate model performance, we employed a five-fold cross-validation approach in the training cohort including training and validation set. During the partitioning of the training and testing cohort, the distribution of data within each class was maintained proportionally. This ensured that data originating from a single patient was not split across different cohorts, thereby preserving the integrity of the experimental results. To mitigate the risk of model overfitting, we implemented optimal data augmentation techniques on the training cohort, predominantly including random spatial transformations, non-rigid deformations, and noise injections, while no augmentations were applied to the testing cohort. To address the issue of class imbalance, all network models involved in comparative experiments consistently employed class-balanced sampling during the training process. This entailed the sampler first selecting a class at random, followed by the random selection of a sample from that class for output.

In our study, training was conducted utilizing the Focal Loss with a class-balancing factor. This loss function dynamically addresses the challenges stemming from data imbalance. The definition of the loss is as follows:

$$L_{CB\_focal}\left(Z,C\right)=-\frac{1-\beta }{1-\beta^{n_{c}}}\sum_{i=1}^{C}{\left(1-p_{i}^{t}\right)}^{\gamma }\log\left(p_{i}^{t}\right)$$

γ = 2.0, β = 0.9999, $n_{c}$ represents the number of samples in category C , C represents all categories amd $C\in \left\{\text{RCC}-12,\text{RCC}-34\right\}$ ,The predicted output is $Z={\left[z_{RCC-12},z_{\text{RCC}-34}\right]}^{⊺}$ .

All models used in this experiment were constructed using the PyTorch framework and trained on an NVIDIA RTX3090 GPU. The SGD optimizer was employed with an initial learning rate of 0.05, which was halved every ten epochs. Model training was performed for 100 epochs across B-mode, CEUS-mode, and a fused B + CEUS modality, utilizing a five-fold cross-validation approach in the training cohort. In each round of five-fold cross-validation, models based on B-mode, CEUS-mode, and B+ CEUS mode were trained for 100 epochs, respectively, and the models with the highest accuracy on the testing cohort were saved.

### 2.7 Statistical analysis

All statistical analyses were performed using the SciPy package in Python version 3.8. Depending on whether the data conformed to a normal distribution, continuous variables were compared using either Student’s t-test or the Mann-Whitney U test. Non-ordered categorical variables were compared using the chi-square test. Receiver operating characteristic (ROC) curve analysis was employed to evaluate single-modality networks and MUF-Net. Additionally, various metrics were used to assess model performance from multiple perspectives, including sensitivity, specificity, positive predictive value (PPV), and negative predictive value (NPV). Comparison of the difference between areas under the ROC curve (AUCs) was performed using the Delong test. A two-sided *P* value <0.05 was considered statistically significant.

## 3 Results

### 3.1 Patient characteristics

The high-grade group of patients exhibited a median albumin level of 45.30 g/L, compared to 42.00 g/L in the low-grade group. The difference in albumin levels between the two groups was statistically significant (*P* < 0.05). However, other parameters, including gender, age, lesion size, clinical manifestations, and additional laboratory metrics, showed no significant differences between the groups (Table 1).

**TABLE 1:** Patient characteristics.

Clinical data	Low grade (N = 74; n = 3,573)	High grade (N = 26; n = 2,720)	*P* Value
Gender n (%)			0.244
Female	17 (23.0)	9 (34.6)	
Male	57 (77.0)	17 (65.4)	
Age (years)	58 ± 13	54 ± 15	0.231
Tumor max diameter (cm)	4.00 (3.00.6.00)	4.00 (3.00.6.63)	0.734
BMI (kg/m^2^)	23.31 ± 1.72	22.98 ± 2.02	0.435
Clinical manifestations n (%)			0.216
Yes	36 (48.6)	9 (34.6)	
No	38 (51.4)	17 (65.4)	
Surgery n (%)			0.955
Radical nephrectomy	46 (62.2)	16 (61.5)	
Partial nephrectomy	28 (37.8)	10 (38.5)	
Hemoglobin (g/L)	132.93 ± 16.20	140.04 ± 15.29	0.054
Albumin (g/L)	42.00 (39.88, 4.93)	45.30 (41.65, 49.00)	0.023
Alkaline phosphatase (U/L)	78.84 ± 23.33	85.81 ± 26.91	0.211
Lactate dehydrogenase (U/L)	185.61 ± 38.11	182.21 ± 36.26	0.693
total calcium (mmol/L)	2.26 ± 0.14	2.29 ± 0.11	0.184
WBC (10^9^/L)	6.34 (5.24.8.46)	7.05 (5.88.9.64)	0.098
RBC (10^12^/L)	4.32 ± 0.51	4.48 ± 0.45	0.156
Platelet count (10^9^/L)	187.12 ± 71.84	175.46 ± 49.26	0.446

N, Refers to the number of patients; n, Refers to the number of images.

### 3.2 Classification performance of MUF-Net and other methods

**FIGURE 3:**
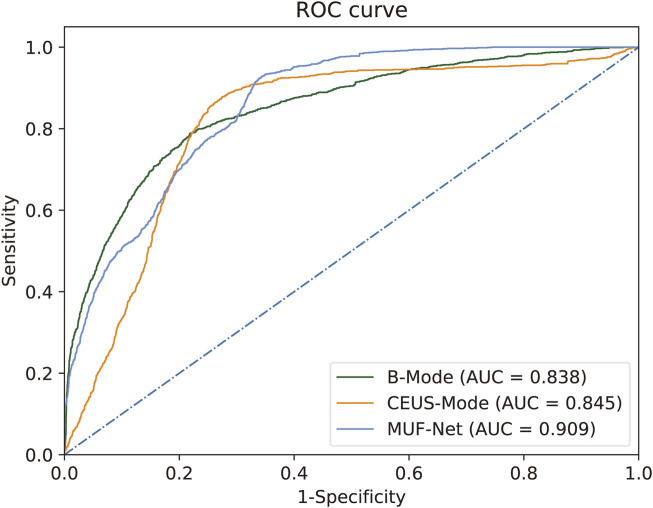
ROC for RCC histological grading by MUF-Net and single-modality models. The multimodal MUF-Net outperformed the unimodal approaches.

**FIGURE 4:**
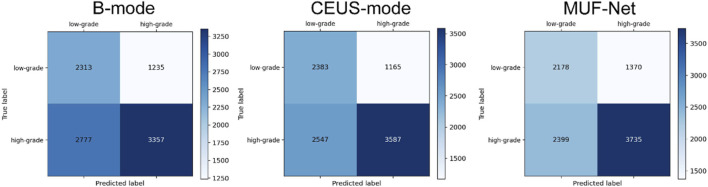
Confusion matrix for single-modality and MUF-Net models.

We evaluated the classification performance of four distinct methodologies in the histological grading of RCC (Table 2). The ROC curves and confusion matrices are shown in Figures 3, 4, respectively.

**TABLE 2:** Performance of various models in the pathological grading of RCC.

Model	AUC (95% CI)	Accuracy (95% CI)	Sensitivity (95% CI)	Specificity (95% CI)	PPV (95% CI)	NPV (95% CI)
B-mode	0.838 (0.689,0.988)	0.808 (0.530,0.857)	0.778 (0.596,0.960)	0.827 (0.779,0.875)	0.750 (0.640,0.860)	0.850 (0.742,0.959)
CEUS-mode	0.845 (0.745,0.944)	0.818 (0.750,0.886)	0.802 (0.645,0.958)	0.825 (0.751,0.898)	0.768 (0.697,0.839)	0.863 (0.780,0.946)
MUF-Net	0.909 (0.829,0.990)	0.859 (0.789,0.928)	0.851 (0.736,0.966)	0.860 (0.791,0.930)	0.816 (0.737,0.895)	0.893 (0.825,0.961)

When EfficientNet-B3 was trained solely with B-mode images, the model achieved an AUC of 0.838, an accuracy of 0.808, a sensitivity of 0.778, a specificity of 0.827, a PPV of 0.750, and an NPV of 0.850.

With training on CEUS-mode images only, EfficientNet-B3 achieved an AUC of 0.845, an accuracy of 0.818, a sensitivity of 0.802, a specificity of 0.825, a PPV of 0.768, and an NPV of 0.863.

When jointly trained on B + CEUS fused modality images using MUF-Net, the model yielded an AUC of 0.909, an accuracy of 0.859, a sensitivity of 0.851, a specificity of 0.860, a PPV of 0.816, and an NPV of 0.893.

### 3.3 Heatmaps of model predictions

**FIGURE 5:**
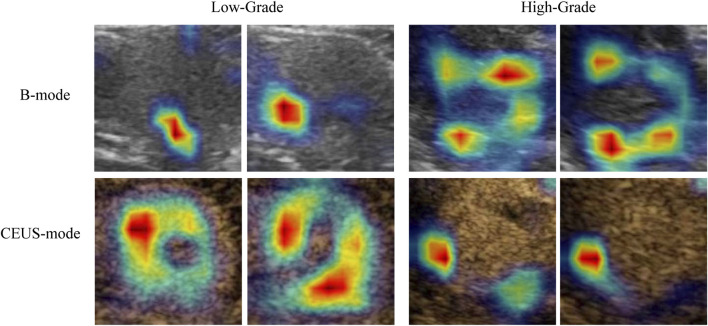
Grad-CAM visualization of the model’s focal regions and morphology within RCC lesions across different groups. The red color represents higher weights.

The Grad-CAM-generated heatmaps in Figure 5 illustrate the regions that are most influential to the model’s predictions for different grades of RCC lesions. In both the low-grade (G1-G2) and high-grade (G3-G4) groups, Grad-CAM provides an intuitive visualization of how the model integrates information from different ultrasound modalities, including B-mode and contrast-enhanced ultrasound (CEUS). For ultrasound images of the same lesion within the same grade group but from different modalities, Grad-CAM effectively visualizes the specific locations and morphologies that the model focuses on, providing a visual explanation of its decision-making process (color-coded, with red indicating a higher degree of contribution). Regardless of whether the lesion is in the low-grade or high-grade group, the heatmaps reveal complementary features across the dual-modality images of the same lesion. In the low-grade group, the CEUS modality contributes more significantly to the region of interest (ROI) compared to B-mode. Conversely, in the high-grade group, B-mode focuses on a greater area compared to the CEUS modality.

## 4 Discussion

60% ∼ 85% of RCC is ccRCC (Linehan and Ricketts, 2019; Inamura, 2017), patients with ccRCC usually lack clinical symptoms in the early stages, and 25% ∼ 30% of patients have metastases at diagnosis (Padala et al., 2020). The nuclear grading of RCC serves as a crucial indicator of invasiveness and represents a significant independent prognostic risk factor for patients with RCC (Galtung et al., 2022; Novara et al., 2007). CEUS provides real-time, continuous, and dynamic observations of renal tumor perfusion, reducing the risk of missing diagnostic information compared to interval-based scans with CECT and CEMRI (Xue et al., 2013; Huang et al., 2023; Velasquez-Botero et al., 2022). In this study, we developed the MUF-Net model, which integrates features from B-mode ultrasound and contrast-enhanced ultrasound mode, successfully achieving non-invasive classification of renal cell carcinoma at nuclear grading of differentiation.

To date, only three studies have utilized ultrasound images for RCC grading, underscoring the uniqueness of our research (Bai et al., 2024; Yang et al., 2024; Luo et al., 2024). The AUC values reported in these previous studies ranged from 0.785 to 0.852, while our model achieved an AUC of 0.909, demonstrating superior diagnostic performance. Our study differs from the existing research in several significant aspects. First, whereas one study used ultrasound radiomics combined with clinical data for Fuhrman grade prediction (Yang et al., 2024), our deep learning approach bypasses manual feature extraction by directly leveraging multimodal data, thereby enhancing both accuracy and generalizability. Second, compared to the CEUS-only XGBoost model (Luo et al., 2024), our multimodal MUF-Net model integrates B-mode and CEUS, capturing complementary features that boost prediction accuracy. Lastly, unlike the RepVGG-based CEUS deep learning model (Bai et al., 2024), which relied solely on CEUS, our fusion of structural (B-mode) and vascular (CEUS) data offers a more comprehensive differentiation of ccRCC grades, ultimately leading to improved diagnostic efficacy and greater clinical utility. Most prior studies have focused on CT or MRI-based models, with variable outcomes. Zhao et al. developed a deep learning model based on MRI, achieving an accuracy of 88% and an AUC of 0.88 in the testing set, with slightly lower performance in the validation set (Zhao et al., 2020). Other studies, such as those by Shu et al. and Lin et al., explored machine learning models using texture features from CECT or CT scans, with AUCs ranging from 0.719 to 0.87 (Shu et al., 2019; Lin et al., 2019). In comparison, our multimodal fusion model, MUF-Net, which integrates B-mode and CEUS-mode data, demonstrated superior performance, achieving an accuracy of 85.9% and an AUC of 0.909. Even when using individual modalities (B-mode or CEUS-mode), our model’s performance (AUCs of 0.838 and 0.845, respectively) surpassed most previous research.

The fusion of B-mode and CEUS-mode features in MUF-Net boosts the model’s performance in RCC grading tasks, validating our previous hypotheses. This can be attributed to the complementary characteristics between ultrasound B-mode and CEUS-mode. B-mode offers clear visualization of tumor size and morphological features, while the CEUS-mode reveals tumor heterogeneity through the microcirculation perfusion process. This complementarity enables MUF-Net to integrate richer information by extracting and fusing features from both modalities, thus enhancing the accuracy of tumor identification. This is an innovative aspect of this study. Furthermore, the fused multi-modal features of MUF-Net are based on the cross-modality feature relationships within the tumor. This means that the model associates feature from different ultrasound modalities that correspond to the same spatial locations. Such feature associations help mitigate the interference of irrelevant and noisy features, allowing the model to focus more effectively on the key characteristics of the tumor. This further enhancement in model performance represents another innovative contribution to our research.

When comparing the performance of convolutional neural network (CNN) models individually trained on B-mode and CEUS-mode, it was evident that CEUS-mode yielded superior results. This enhancement is likely due to abundant biological information regarding tumor characteristics contained within the CEUS data, enabling the model to capture more relevant information associated with tumor pathological grading. Additionally, the 95% confidence interval (95% CI) for models trained on CEUS-mode was narrower than that of B-mode, suggesting that CEUS-trained models exhibit greater stability and robustness, thereby providing more reliable diagnostic outcomes. Notably, in the validation set, an improvement of over 6% in AUC is observed, along with significant enhancements in other evaluation metrics.

Notably, the heatmaps generated by Grad-CAM also provide an intuitive interpretation of the model’s predictions, highlighting distinct focus regions for different grading groups. For lesions within the same grading category, Grad-CAM delineates specific locations, morphologies, and the significance of these areas for classification. The focus regions are complementary between B-mode and CEUS-mode images, with low-grade tumors predominantly emphasized in CEUS, while high-grade tumors are more focused on B-mode. Currently, there is a lack of studies quantifying these image features. Fan et al. partially explained that CEUS features are effective in differentiating high-grade from low-grade tumors. They found that the presence of incomplete pseudocapsules was significantly higher in high-grade tumors, suggesting that certain CEUS characteristics correlate with tumor aggressiveness and grade (Fan et al., 2024). Our findings are similar to previous studies in demonstrating that machine learning and deep learning models effectively differentiate between low-grade and high-grade renal cell carcinoma (RCC). Specifically, deep learning models outperform traditional methods, providing a robust foundation for AI-driven research on pathological subtypes and molecular classifications of renal tumors. This has the potential to significantly enhance clinical decision-making in the management of renal cancer.

## 5 Limitations

While the MUF-Net model demonstrated promising classification performance, several limitations should be acknowledged One limitation of our study is the relatively small sample size, particularly the low proportion of high-grade RCC cases, which may impact the model’s generalizability. While we used a large number of ultrasound images, the limited patient sample size may reduce the model’s robustness and its ability to generalize to new data, especially from diverse populations or institutions. To address this, we employed data augmentation techniques such as rotation, scaling, and flipping to increase data diversity, enhancing the model’s robustness. Additionally, class-balanced sampling and weighted loss functions were used to mitigate class imbalance, ensuring fair performance on both low-grade and high-grade RCC cases. Nevertheless, larger and more diverse datasets, particularly with a balanced distribution of RCC grades, are needed for improved generalizability. Future studies with multicenter datasets would help enhance the model’s clinical applicability and robustness across various clinical settings. On the other hand, self-attention and cross-attention mechanisms could be employed to facilitate direct interactions among cross-modality features in the future, thereby further improving performance.

## 6 Conclusion

The MUF-Net model, utilizing multimodal data from grayscale ultrasound and CEUS videos, demonstrates the feasibility of automatic nuclear grading for RCC, achieving excellent classification performance.

## Data Availability

The original contributions presented in the study are included in the article/supplementary material, further inquiries can be directed to the corresponding authors.
